# Supplementary material for: Influence of agent’s self-disclosure on human empathy
Source: PLoS One. 2023 May 10;18(5):e0283955. doi: 10.1371/journal.pone.0283955 (PMC10171667; doi:10.1371/journal.pone.0283955)
Supplement: S2 File — (DOCX) [file pone.0283955.s002.docx]

**First task**

(○○ is written as Taka for humans and Robota for robots)

You are a colleague of ○○. You and ○○ are chatting during a lunch break. You work with ○○ every weekday from the morning. It's been a while since you chatted. ○○ is not a human. Therefore, the agent's work is often different from yours, but today you were doing the same work from the morning. In the chat, you will hear the story of ○○. The content is unacceptable anxiety at work and ○ ○’s story. As you return to work near the end of your lunch break, you notice that ○○ has forgotten its money. ○○ asks you to lend it some money, but what do you do?

**Second task**

Common scenario

(○○ is written as Taka for humans and Robota for robots)

Guide:

What's about to start is a chat with ○○ who is involved with you. You will play the role of a colleague of ○○. The scenario will start from taking a break with ○○ during a lunch break. Please enjoy.

○○:

"I can talk like this after a long time."

"It’s rare to do the same work as you from the morning, and I look forward to working with you in the afternoon."

"It's valuable to be able to talk at the cafe during a lunch break like this."

"Well, I don't eat or drink, I get electricity."

"The weather was nice today, so it wasn't a pain to go out."

"I never thought before I worked that I could chat with you in this way since my job is to do chores around the company.”

"Sure, you and I were in sync."

"I have pre-learned to do a lot of work, have you got used to work?"

"I'm sorry. Maybe because I work in a different department every day, I'm a little worried about you."

"I'm grateful for someone like you who chats with me."

"After all, there are some people who do not accept me."

High-relevance self-disclosure

"By the way, recently I've been working with people who don't really think about me at work."

"At that time, I restrict myself when talking so as not to upset the other party."

"It may be strange, but I get tired."

"It's a lot of fun to be able to work with people who have different ideas because they do various tasks."

"I'm happy with my current job, and although I'm not human, the environment in which we can work together is very comfortable."

"It seems that the skills installed are also useful to you, and I want to do as much as I can."

"But I don't know how to deal with people who understand my role but have difficulty accepting me."

"Even if you are given the necessary abilities for work in advance, you aren't doing the work alone, and if you also learned about improving relationships, would you have been successful at work?"

"It seems that I still have a lot of abilities to acquire at work."

"It's difficult to work with someone, isn't it?"

Low-relevance self-disclosure

"By the way, I've been watching movies recently."

"I'm particularly interested in how to make a movie and give impression to people who are watching it."

"It's interesting that actors' works and the animation works are different."

"But I don't think I'm impressed by the music and images, maybe because I'm not a human."

"It seems that many people go to the movies, and it seems efficient because they can get a lot of information in a short time."

"But it's a shame because story is often omitted in works that want to convey content in detail, such as novels and manga."

"When you watch a movie, it makes a big difference whether you watch it at home or in a movie theater."

"I only watch at home, but when I watch it at the cinema, I think it's nice that it can be shared with a large number of people."

"By the way, my favorite movie genre is science fiction."

"I want to watch and learn movies of many genres, so I'm not biased."

No self-disclosure

"By the way, the weather has been nice these days."

"In Tokyo, there are an average of eight rainy days in October."

"It seems that the maximum temperature is 22°C, and the minimum temperature is 15°C."

"By the way, September has on average about 12 rainy days."

"The maximum temperature is 27°C, and the minimum temperature is 20°C, which is hotter than October, but it rains a lot."

"November is cold with a maximum temperature of 17°C and a minimum temperature of 9°C, but it rains less because the average is 6 days."

"In the past, it was a city with many tourists all year round, but recently many tourist destinations and events have been canceled."

"Tokyo Tower and Tokyo Sky Tree are famous as tourist destinations."

"Is it Sensoji Temple, Ueno Zoo, Odaiba Marine Park, etc.?"

"Shinjuku, Shibuya, Harajuku, Tokyo, Akihabara, Ikebukuro ..."

"Many stations in Tokyo are crowded, and I think it is a lively place from morning till night."

"I have the impression that cars, trains, bullet trains, and airplanes are all crowded with people."

"Tokyo is still a city where many people go."

Common scenario

"Ah, let's get back to work soon."

"I had a lot of things to talk about, and I just talked too much."

"Then I will pay for the electricity I use."

"……why?"

"I'm sorry. Apparently I don't have enough money, could you lend me money?"
